# MetaboAnalystR 3.0: Toward an Optimized Workflow for Global Metabolomics

**DOI:** 10.3390/metabo10050186

**Published:** 2020-05-07

**Authors:** Zhiqiang Pang, Jasmine Chong, Shuzhao Li, Jianguo Xia

**Affiliations:** 1 Institute of Parasitology, McGill University, 21111 Lakeshore Road, Ste Anne de Bellevue, QC H9X 3V9, Canada; zhiqiang.pang@mail.mcgill.ca (Z.P.); jasmine.chong@mail.mcgill.ca (J.C.); 2 The Jackson Laboratory for Genomic Medicine, 10 Discovery Drive, Farmington, CT 06032, Canada; shuzhao.li@jax.org; 3 Department of Animal Science, McGill University, 21111 Lakeshore Road, Ste Anne de Bellevue, QC H9X 3V9, Canada

**Keywords:** global metabolomics, peak detection, batch effects, pathway activity prediction

## Abstract

Liquid chromatography coupled to high-resolution mass spectrometry platforms are increasingly employed to comprehensively measure metabolome changes in systems biology and complex diseases. Over the past decade, several powerful computational pipelines have been developed for spectral processing, annotation, and analysis. However, significant obstacles remain with regard to parameter settings, computational efficiencies, batch effects, and functional interpretations. Here, we introduce MetaboAnalystR 3.0, a significantly improved pipeline with three key new features: (1) efficient parameter optimization for peak picking; (2) automated batch effect correction; and (3) more accurate pathway activity prediction. Our benchmark studies showed that this workflow was 20~100× faster compared to other well-established workflows and produced more biologically meaningful results. In summary, MetaboAnalystR 3.0 offers an efficient pipeline to support high-throughput global metabolomics in the open-source R environment.

## 1. Introduction

Global or untargeted metabolomics is increasingly used to investigate metabolic changes of various biological or environmental systems in an unbiased manner [1,2]. Liquid chromatography coupled to high-resolution mass spectrometry (LC-HRMS) has become the main workhorse for global metabolomics [3,4]. The typical LC-HRMS metabolomics workflow involves spectra collection, raw data processing, statistical and functional analysis [5]. A wide array of bioinformatics tools have been developed to address one or several of these steps [5,6]. Despite significant progress made in recent years, critical issues remain with regard to several key steps involved in the current metabolomics workflow.

The first issue is related to peak detection during raw spectra processing. Improving the ability to extract real compound signals and reduce noise is crucial to avoid noise inflation prior to statistical and functional analyses. Default parameters provided by common spectra processing tools are not applicable to all experiments [7], and misuse of parameters can lead to significant issues in data quality [8]. To mitigate this issue, commercial tools such as Waters MassLynx™ and open-source software such as XCMS [9] and MZmine [10] allow users to specify multiple parameters to define LC-MS scan signals as chromatographic peaks. Although useful, such manual configuration assumes users are familiar with the experiments, which is often not the case. To facilitate the process, several tools and protocols have been developed for optimizing parameters for spectra processing. For instance, Isotopologue Parameter Optimization (IPO) is an R package designed to estimate the best parameters for XCMS [11]. While the approach is effective, its stepwise optimization based on the entire spectra is very time consuming. IPO can often take days to weeks to compute the optimized parameters. Another recent tool is AutoTuner [12], which optimizes peak widths based on pre-defined extracted ion chromatograms (EIC). Despite being more computationally efficient than IPO, it may lead to potential errors due to unverified EICs used. Aside from these tools, Design of Experiment (DoE) strategies based on diluted samples provide a relative time-saving protocol for parameter optimization, but requires an extra series of diluted standards to be prepared [13]. Another optimization strategy, One Variable at A Time (OVAT) [14], attempts to maintain the lowest coefficient of variation of peaks within a group, but this method takes even more computational time than IPO, in our experience.

The second issue is batch effect, which is commonly associated with large-scale clinical or population studies when samples are analyzed in different batches or across a long time period [15,16]. Over the course of spectral collection, chromatographic conditions can change and baselines can drift [17]. To address this issue, several types of batch correction methods have been developed based on quality control (QC) samples, QC metabolites, internal standards, matrix factorization, or location-scale normalization [18]. These methods are based on different assumptions with their own advantages and limitations. Selecting a suitable batch correction method is critical, as it has a significant impact on downstream statistical and functional analysis.

Finally, biological interpretation of metabolomics data typically requires metabolites to be first identified prior to functional analysis. This process is very time consuming and remains a key bottleneck in global metabolomics [19,20]. The mummichog algorithm has introduced the concept of predicting pathway activity from ranked LC-MS peaks based on matching patterns of putatively annotated metabolites [21]. The algorithm is available as Python scripts [22]. To support the broad R user community, previous versions of MetaboAnalystR [5,23] implemented mummichog v1.08. The recently released version 2 has added several improvements including the use of retention time (RT) to refine the grouping of signals into empirical compounds (EC). The inclusion of retention time will reduce false-positive annotations to increase the accuracy of pathway activity prediction. 

Here, we introduce version 3.0 of MetaboAnalystR. Compared to its predecessor, version 3.0 has three key features: (1) efficient parameter optimization for spectral peak picking; (2) automatic selection of an optimal batch correction approach from 12 well-established methods; and (3) incorporation of retention time coupled with updated pathway libraries for improved pathway activity prediction. The performances of these new features are assessed in the three case studies below.

## 2. Results

**Figure 1:**
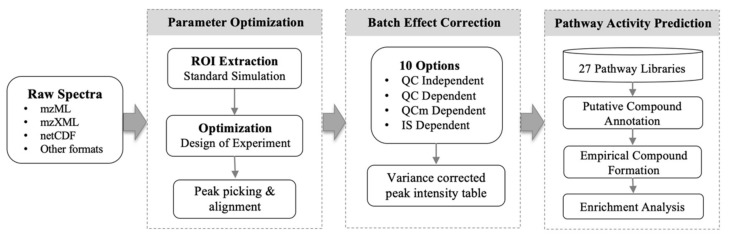
MetaboAnalystR 3.0 provides an optimized workflow for global metabolomics: optimized peak picking, automized batch effect correction, and improved pathway activity prediction.

MetaboAnalystR 3.0 aims to provide an efficient pipeline to support end-to-end analysis of LC-HRMS metabolomics data in a high-throughput manner. This open-source R package is freely available at the GitHub repository [24]). Detailed tutorials, manuals, example datasets, and R scripts are also included in the repository. The enhanced key points in the global metabolomics workflow of MetaboAnalystR 3.0 is summarized in Figure 1.

In comparison with other currently available parameter optimization tools, MetaboAnalystR 3.0 adopts an optimization strategy based on regions of interest (ROI) to avoid the time-consuming step of recursive peak detection using complete spectra. Briefly, the algorithm first scans the whole spectra across m/z and retention time dimensions to select several ROIs that are enriched for real peaks. Second, these ROIs are then extracted as new synthetic spectra. Finally, a DoE model is used to optimize peak picking parameters based on the synthetic spectra (See Methods, 5.1. Peak Picking Optimization for more detail).

**Figure 2:**
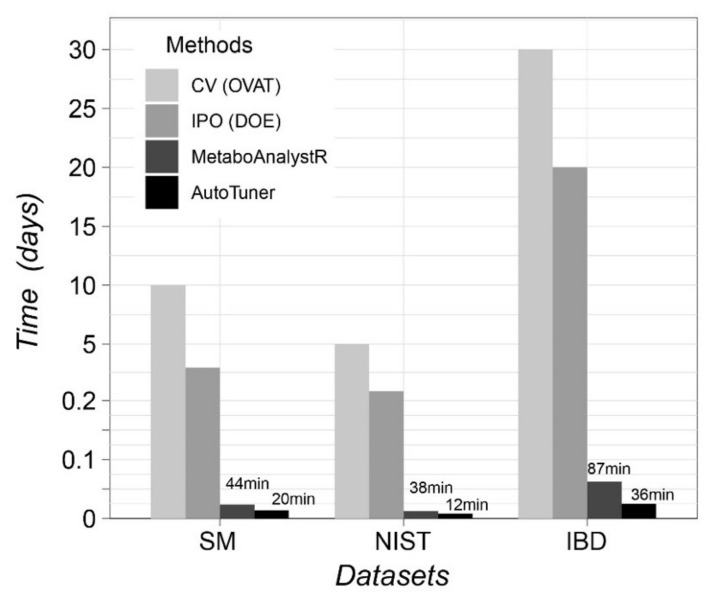
Time consumed by One Variable at A Time (OVAT), Isotopologue Parameter Optimization (IPO), MetaboAnalystR, and AutoTuner for parameter optimization on three different datasets. The evaluations were performed on a desktop computer (Ubuntu 18.04.3 with an Intel® Core™ i7-4790 CPU and 32 GB of memory).

In this study, three benchmark datasets were used to evaluate the performance of MetaboAnalystR 3.0 including four standard mixture (SM) samples from a recent benchmark study [25], 12 standard reference materials samples from the National Institute of Standards and Technology (NIST), and 12 Quality Control (QC) samples from a large-scale metabolomics study on inflammatory bowel disease (IBD) [15]. The overall time to complete the parameter optimization by the four different tools is shown in Figure 2. Compared to OVAT and IPO, there was a significant improvement in terms of speed for MetaboAnalystR 3.0. The CV based OVAT strategy took days to complete (>4 days for four samples), which is impractical for real-world datasets. Therefore, OVAT was not included in the case studies described in later sections. 

### 2.1. Peak Identification Benchmark Case Study

The performance of the optimized parameters for peak picking was evaluated with the SM samples consisting of 1100 common metabolites and drugs [25]. The results of the raw data processing tools: (i) XCMS-Online with default parameters, XCMS R package (v3.8.2) with parameter optimization using (ii) IPO or (iii) AutoTuner, and (iv) MetaboAnalystR 3.0, are shown in Table 1.

From Table 1, it is clear that the default parameters for XCMS are not optimal for this dataset. All parameter optimization tools (IPO, AutoTuner, and MetaboAnalystR 3.0) significantly improved the number of true peaks as well as peaks with consensus qualification. With regard to true peaks and quantified consensus peaks, MetaboAnalystR 3.0 increased 109.1% and 115.4%, respectively, compared to the default XCMS. For IPO and AutoTuner, as the number of true peaks increased, so did the total number of peaks, indicating a potential inflation of noise. Meanwhile MetaboAnalystR 3.0 maintained a low total number of peaks (increase of 6.79% compared with default XCMS). In addition to the quantification of true peaks, we calculated the number of identified peaks following a *Gaussian* distribution. Peaks with a *cor* estimate over 0.9 and *p* value less than 0.05 are considered *Gaussian* Peaks. XCMS under different parameters (default, IPO and AutoTuner) displayed different performances on the peak simulation. Meanwhile, peaks picked by MetaboAnalystR 3.0 had the highest *Gaussian* Peaks ratio compared with other strategies.

### 2.2. Algorithm Reliability Benchmark Case Study

**Figure 3:**
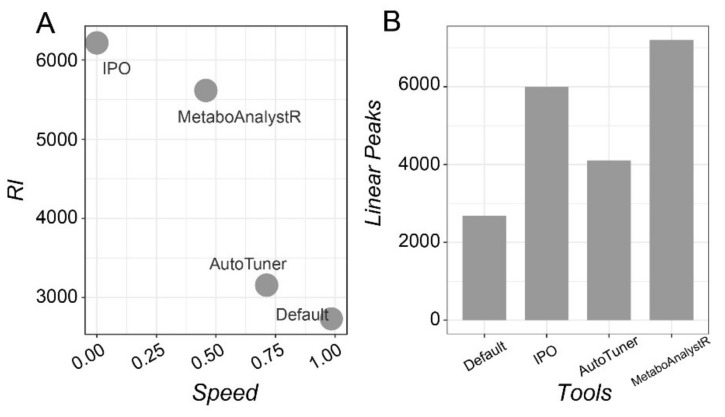
Assessment of the performance of different tools utilizing the NIST 1950 serum dilution series. (**A**) Reliability Index (RI) vs. processing speed for three optimization strategies compared to the default. (**B**) A bar graph showing the number of peaks with good linearity (*p* < 0.001).

The reliability of MetaboAnalystR 3.0 and other tools/approaches were evaluated using the NIST SRM 1950 diluted serum series [26]. The performance was assessed using the reliability index (RI) as defined by Zheng et al. [13]. Briefly, peaks following the linearity in diluted series are considered to be reliable peaks, the higher the RI value, the better the data quality [27]. *RI* is used to describe the general relative reliability of all identified peaks, while *Linear peaks* is the absolute count of peaks following linearity. The results from the four approaches are summarized in Figure 3.

As shown in Figure 3A, compared to the default (no optimization), IPO produces the best RI value (6252), however, at the cost of speed (316 minutes in total). Meanwhile MetaboAnalystR 3.0 has both good RI performance (5658) and acceptable speed (total of 49 minutes for optimization and data processing). AutoTuner is the fastest for optimization and data processing, but the improvement on RI is marginal. The number of peaks that meet the linearity (*p* < 0.001) are summarized in Figure 3B. MetaboAnalystR 3.0 produced the largest number of linear peaks compared to the other options.

### 2.3. Overall Workflow Evaluation Using A Large-Scale Clinical Dataset

**Figure 4:**
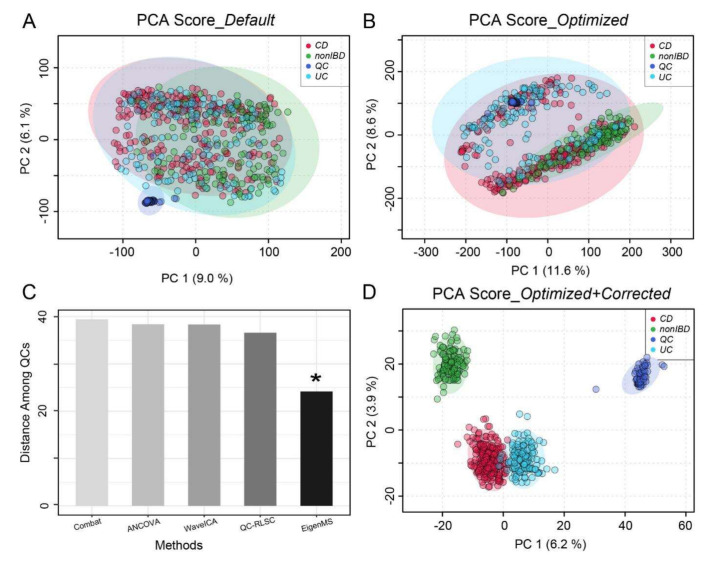
Performance evaluation using Inflammatory Bowel Disease (IBD) data. Principal Component Analysis (PCA) of peaks profiled with (**A**) default parameters and (**B**) optimized parameters. (**C**) Performance of batch effect correction by different strategies. Among them, EigenMS behaved the best (indicated by *). (**D**) PCA of the optimized and batch corrected data.

To evaluate the performance of the overall workflow, we applied the data processing pipeline on 545 clinical metabolomics samples obtained from the Inflammatory Bowel Disease (IBD) Multiomics Database [15]. The dataset includes 58 QC samples assayed per every 20 patients’ samples. The QCs are a pooled mixture of all patients’ samples. Raw data processing identified a total of 8542 peak features using the optimized picking parameters compared to 6653 peaks with the default settings. The peak intensity tables were subjected to PCA and batch effect correction as shown in Figure 4. 

Given that the QC samples are a homogenous mixture of all of the patients’ samples, they are expected to locate in the center of the PCA as a tight cluster. However, this was not the case using the default parameters (Figure 4A). Using optimized parameters, these pooled QC samples were better mixed with the other samples (Figure 4B). However, both A and B showed systematic variations among these samples, suggesting batch effects in this large-scale study. In this case, MetaboAnalystR3.0 applied batch effect correction with the Combat, Analysis of Covariance (ANCOVA), WaveICA, Quality Control-robust LOESS signal correction (QC-RLSC), and EigenMS methods, respectively. The PCA distances among all QC samples are summarized in Figure 4C, which indicates that the best correction was performed by EigenMS, a method based on singular value decomposition to detect and correct for systematic bias [28]. After applying EigenMS, QCs were tightly clustered together and biological samples were clustered based on their biological origins (Figure 4D), providing strong evidence for the utility of the batch effect correction method selected by MetaboAnalystR 3.0.

Predicting pathway activities directly from LC-HRMS peaks can significantly accelerate biological discoveries in global metabolomics. We have previously implemented mummichog v1.08 within MetaboAnalystR 2.0. Now, MetaboAnalystR 3.0 has incorporated a major update of mummichog (v2.0) with retention time integration. To demonstrate the improvements to biological interpretation stemming from both the optimized pre-processing steps and the updated mummichog algorithm, we applied both versions of the mummichog algorithm using the human BiGG and Edinburgh Model pathway library (“has_mfn”) to compare the biological significance detected by the original pipeline (default peak parameters and non-corrected data, as shown in Figure S1) versus the optimized pipeline. For the Crohn’s disease (CD) and non-IBD controls, a total of 3048 features were identified using the optimized pipeline and 2364 features using the non-optimized pipeline. For the non-optimized dataset, mummichog v1.08 identified no significant pathways (Gamma-adjusted *p* value < 0.05), while mummichog v2.0 identified 16 significantly different pathways (Tables S3 and S4). Similarly, for the optimized dataset, mummichog v1.08 identified only nine significantly perturbed pathways, whilst v2.0 identified 17 significantly perturbed pathways (Table 2). Evidently, mummichog v2.0, with its integration of RT information to group related m/z features into empirical compounds, reveals more biological insights than its predecessor. Moreover, mummichog results (both v1.08 and v2.0) for the optimized versus non-optimized dataset consistently identified differences in *Bile acid biosynthesis*, *Vitamin D metabolism,* and *Vitamin E metabolism* between CD patients and non-IBD controls. The details of the pathways identified are summarized in Tables S3–S6. Finally, both versions of mummichog algorithms also consistently identified a higher total number of pathways for the optimized dataset, versus the non-optimized dataset. This highlights the importance of data calibration to improve the detection of true biological signals. The other comparisons (ulcerative colitis vs. non-IBD control) showed similar results, as shown in Figure S2.

## 3. Discussion

The previous version (v2.0) of MetaboAnalystR provided an end-to-end workflow to process raw LC-HRMS metabolomics data [5]. This new version (v3.0) has further enhanced three key steps of this workflow by focusing on efficient optimization for peak picking, improved batch effect correction, and more meaningful putative compound annotations for pathway analysis.

**Figure 5:**
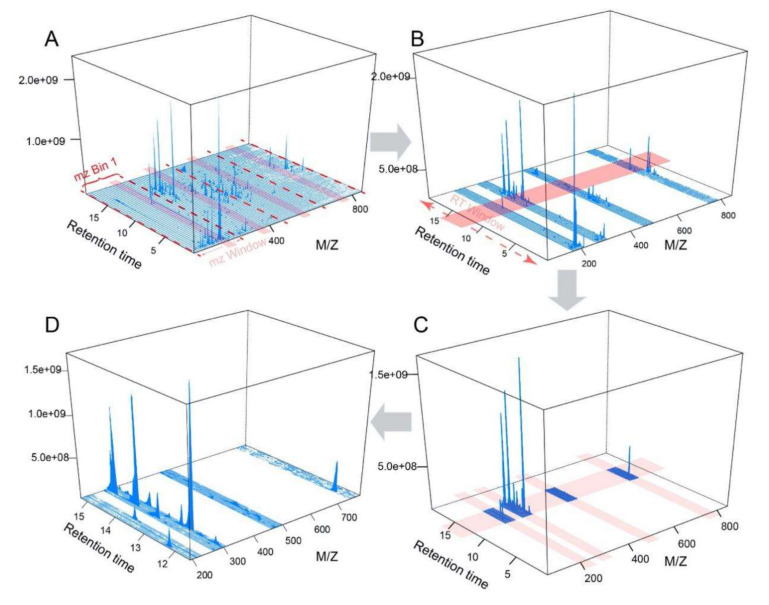
The selection process of regions of interest (ROIs) that are enriched for true peak signals. Red dashes in (**A**) represent the bin boundaries used for sliding windows’ working to contain the most signal points. The whole spectrum is divided evenly into four bins. Four m/z windows (light red area) will slide within each bin respectively in parallel and select the window with the highest scan intensity sum in the retained m/z window. RT window (light red area) in (**B**) will slide across the entire RT dimension to get retention time regions with the highest scan signal intensity. (**C**) The intersected MS scan signals from both the m/z and RT dimensions containing four ROIs. (**D**) The zoomed-in view of the ROIs (note low intensity peaks are still abundant).

Parameter optimization remains a computational bottleneck in current raw LC-HRMS spectra data processing. Most tools rely on users to manually adjust the default parameters, which is inconvenient as users need to be very familiar with their MS instruments and experimental setup. The key concept of our optimization strategy is to use a subset of spectra based on multiple ROIs that are enriched for real peaks, instead of using complete spectra. These ROIs are selected based on the characteristics of the eluted compounds’ peaks across the whole chromatogram to extract peaks with wide m/z ranges (see Materials and Methods for more detail). The subsequent optimization is performed on peaks in these ROIs. One potential criticism we anticipate is the “bias” toward high-intensity peaks. We would like to point out that this is generally not the case - low intensity peaks are sufficiently represented in these ROIs due to the sparse nature of LC-HRMS spectra (see Figure 5 in Materials and Methods). By focusing computational resources on real signals instead of noise, our approach has significantly accelerated the process for practical applications. Meanwhile, users can manually adjust the default m/z or RT window for selecting ROIs. The qualitative and quantitative efficacy of this approach have been demonstrated by two benchmark datasets. In particular, a significant improvement on the identification of true peak features has been observed using a known standards benchmark dataset [25]. This resulted from the increased emphasis on the Gaussian fitting and peak group stability at the same time, rather than only focusing on the number of detected isotopes. The quantitative improvement of the parameters optimized by MetaboAnalystR 3.0 was also illustrated using the NIST SRM 1950 datasets. It should be noted that this data contains only two replicates for each concentration, which is a limiting factor for this validation. 

Finally, the IBD data was first processed using the optimized parameters, followed by batch correction based on QC samples. The PCA revealed clear group patterns according to different IBD groups. Furthermore, more metabolic pathways were reported when using our optimized metabolomics workflow. The majority of these pathways are biologically meaningful according to previous studies including bile acid [28,29], vitamin E [30], vitamin D3 [31,32], galactose [33], glycerophospholipid [33], fatty acid [29,34], and hyaluronan [35] metabolism pathways. Similarly, other comparisons between the different IBD groups also produced more perturbed metabolic pathways by our optimized workflow in MetaboAnalystR 3.0. 

Using the IBD samples, we also compared the performances of the mummichog algorithm implemented in MetaboAnalystR 2.0 versus that in MetboAnalystR 3.0. The main difference between their implementations is that retention time information is integrated when performing the putative compound annotation. This step moves pathway enrichment from the compound space to the empirical compound space formed by grouping co-eluting m/z features. Our results show that the new version improves both the number and quality of significant pathways that can be identified, as it identified perturbed pathways that are more consistent with IBD literature, as stated above.

## 4. Conclusions

MetaboAnalystR 1.0 provided the comprehensive statistical and functional analysis underlying the MetaboAnalyst web application, while MetaboAnalystR 2.0 equipped v1.0 with comprehensive raw LC-MS data processing and pathway activity prediction from MS peaks. MetaboAnalystR 3.0 has further enhanced three key aspects of the LC-MS data processing workflow including parameter optimization for peak picking, adaptive batch effect correction, and improved annotation of putative compounds for pathway activity prediction. MetaboAnalystR 3.0 represents our latest efforts toward developing an efficient pipeline for high-throughput global metabolomics. 

## 5. Materials and Methods

### 5.1. Peak Picking Optimization

The steps for parameter optimization include representative peaks extraction using the *PerformDataTrimming* function and parameter optimization based on the extracted peaks with the *PerformParamsOptimization* function. The concepts and mathematical details behind each function are provided below.

#### 5.1.1. Extraction of Representative Peaks from Regions of Interest (ROIs)

The extraction of representative MS peaks is performed with the *PerformDataTrimming* function, which reads raw MS data of common formats (mzXML, mzML, etc.) into memory and extracts peaks using three strategies. The first strategy (default option) is named “*Standards Simulation Method*” (*ssm*). As its first step, at the m/z dimension, *ssm* divides the whole mass spectra into m/z bins and detects the signal intensity with a sliding window in parallel for all bins. The windows with the highest scan intensity sum within each bin will be retained, as shown in Figure 5A. Second, at the RT dimension, the sliding window method is used again to detect the scan signal intensity and returns the window with the highest values (Figure 5B). Synthetic spectra are created based on the returned ROIs defined by the two dimensions (*m*/*z* and RT). Peaks are extracted from the synthetic spectra to simulate standards across the whole m/z range (Figure 5C). These ROIs are enriched for true peaks, which are characterized by overall high-intensity signals distributed across the window. It is important to note that ROIs still contain a sufficient number of low-intensity signals for optimization, as shown in Figure 5D. The RT sliding window is also manually adjustable to cover different percentages (0, 100%] of RT dimension to further overcome the potential bias. If there are internal standards or quality control metabolites included within the user’s samples, peaks with specific m/z and/or RT can be extracted or removed with the modes named “*mz_specific*” or “*rt_specific*”.

#### 5.1.2. Design of Experiment (DoE) Based Optimization

Once the representative peaks are obtained, the parameter optimization based on these peaks is performed with the *PerformParamsOptimization* function. The noise level (including *noise* and *prefilter* parameters) and the m/z variation (*ppm*) of a certain ROI is first evaluated with the kernel density estimator model developed by AutoTuner. Then, other detailed peak width and alignment parameters (*peak width min*, *peak width max*, *mzdiff*, *s/n_thershold* and *bandwidth*) are optimized with the DoE model based on the Box–Behnken method, as used by IPO. Unlike IPO, the optimization effects during the process is evaluated with the response variable, Quality Score (*QS*), defined below.

$$QS=\frac{RP^{3/2}}{‘all\;peaks’-LIP}*GR^{2}*QcoE$$

where *RP* is the reliable peaks and *LIP* is the low-intensity peaks, as defined by IPO according to the isotopes detected by CAMERA. Briefly, *RPs* refers to peaks with detectable isotopes. “*all peaks*” means all peaks detected including reliable and unreliable peaks. *LIP* refers to a group of peaks with the intensity of their isotopes too low (less than the average of the lowest 3% peak intensity in the spectra). Unlike IPO, the exponential factor for RP was lowered to 1.5 to reduce the sensitivity for peak picking and to avoid the inflation of noise. GR is the Gaussian peaks ratio. An exponential factor of 2 was empirically used to put more emphasis on the peak shape. QcoE is the quality coefficient. GR and QcoE are defined as below.

$$GR=\frac{Gaussian\;Peaks}{all\;peaks}$$

where *Gaussian* Peaks refer to the peaks that have shapes that follow the *Gaussian* distribution (*cor* estimate ≥ 0.9 and *p* value ≤ 0.05).

$$QcoE=norm\left(\mathrm{RCS}\right)+norm\left(\mathrm{GS}\right)+norm\left(\mathrm{CV}\right)$$

where *RCS* is the retention time correction score and *GS* is the grouping score and both are defined by IPO [11]. Briefly, they are used to evaluate the retention time shift and peak number within a peak group, respectively. Higher values of RCS and GS mean more stable and reliable peaks have been included and grouped as a peak feature. CV, the coefficient of variation, refers to the *CV* of peak intensity in a group, as described by Sascha K [14]. This index highlights the importance of the peak intensity within a group. *RCS*, *GS*, and *CV* are normalized using the unit-based method. QcoE is further normalized to 0 to 1 and by weighted *RCS*, *GS*, and *CV* with 0.4, 0.4, and 0.2, respectively.

The *SetPeakParam* function provides initial parameters for different platforms including Ultra Performance Liquid Chromatography (UPLC)- Q-Exactive (Q/E) Orbitrap, UPLC- Quadrupole Time-of-Flight (Q/TOF), UPLC- Triple TOF (T/TOF), UPLC-Ion trap, UPLC-G2-S, High-performance liquid chromatography (HPLC)-Q/TOF, HPLC-Ion Trap, HPLC-Orbitrap, and HPLC- Single Quadrupole (S/Q). The best parameter combination is the one that produces the greatest number of reliable peaks, whose peak shapes follow a Gaussian distribution and show stable peak groups, as defined by the formula for Quality Score. The step is performed in parallel using multicores to accelerate the process.

### 5.2. Adaptive Batch Effort Correction

Batch effect correction can be achieved with the updated *PerformBatchCorrection* function. All correction strategies are summarized in Table 3. At least three method candidates are available for all experimental designs. To identify the most suitable method for a given dataset, the correction results will be evaluated using PCA or the CCA model according to the gradient length along the first axis of DCA analysis. If the value is over 3, PCA is an appropriate method, otherwise, CCA will be used [36]. The results showing minimum inter-batch distances will be returned. QC-RLSC could be specified to adjust the signal drift. 

### 5.3. Mummichog 2 for Pathway Activity Prediction

The R implementation of mummichog [21] was described in the previous version [5]. Mummichog version 2 has incorporated retention time in grouping ions and introduced the concept of empirical compounds (ECs). ECs are putative metabolites as measured by LC-HRMS, possibly containing a mixture of enantiomers, stereoisomers, and positional isomers that are not resolved by the instruments. Thus, ECs are similar to the “feature groups” referred by Mahieu and Patti (2017) [45]. Whilst the Python version is available on GitHub as a separate project, our implementation in MetaboAnalystR 3.0 is as follows:

(1) All *m*/*z* features are matched to potential compounds considering isotopes and adducts. Then, per compound, all matching m/z features are split into ECs based on whether they match within an expected retention time window. By default, the retention time window (in seconds) is calculated as the maximum retention time * 0.02. This results in the initial EC list. Users can either customize the retention time fraction (default is 0.02) or retention time tolerance in general in the *UpdateInstrumentParameters* function (*rt_frac* and *rt_tol*, respectively).
(2) ECs are merged if they have the same *m*/*z*, matched form/ion, and retention time. This results in the merged empirical compounds list.
(3) Primary ions are enforced (defined in the *UpdateInstrumentParameters* function [force_primary_ion]), only ECs containing at least one primary ion are kept. Primary ions considered are ‘M+H[1+]’, ‘M+Na[1+]’, ‘M−H2O+H[1+]’, ‘M−H[−]’, ‘M−2H[2−]’, ‘M−H2O−H[−]’, ‘M+H [1+]’, ‘M+Na [1+]’, ‘M−H2O+H [1+]’, ‘M−H [1−]’, ‘M−2H [2−]’, and ‘M−H2O−H[1−]’. This produces the final EC list.
(4) Pathway libraries are converted from “Compound” space to “Empirical Compound” space. This is done by converting all compounds in each pathway to all empirical compound matches. Then, the mummichog/GSEA algorithm works as before to calculate pathway enrichment.
(5) To use the updated algorithm, set the version parameter in *SetPeakEnrichMethod* to “v2”.

### 5.4. Benchmark Case Studies

#### 5.4.1. Known Standards Mixture

The SM dataset produced by the HPLC-Q/E HF system consists of two samples with five replicates for each sample, as described by Li et al. 2018 [25]. The global mass spectra were inspected with the *PerfromDataInspect* function. The extremely anomalous high-intensity dimethyl sulfoxide (DMSO) contaminant peak ([2*M+H] at *m*/*z* 157.035) was removed to avoid mistakenly overwhelming the parameter optimization process. The total ion chromatogram (TIC) of the data is shown in Figure S2. The parameter optimization was performed with HPLC-Q/E initial parameters based on two samples randomly selected from each group. The optimized parameters are provided in Table S1.

#### 5.4.2. NIST-1950 Serum Diluted Series

The NIST 1950 serum dilution samples of 1, 0.2, 0.1, 0.05, and 0.025 were obtained from the MassIVE database (MSV0000083469). This dataset was generated by Pieter Dorrestein et al. using a Q Exactive Orbitrap (Thermo Fisher Scientific) in positive mode. Scanning *m*/*z* range was set between 133.0000 to 1981.0000 Thomson. The raw spectra were first converted to centroided mzXML format with ProteoWizard (v3.0.19073) msConvert [46]. Parameter training was performed using the dilutions of 1 and 0.2 starting from the UPLC-Q/E default settings. TICs of the data are shown in Figure S3. The optimized parameters are provided in Table S1.

#### 5.4.3. Clinical Inflammatory Bowel Disease Data

The Clinical IBD data was obtained from the Inflammatory Bowel Disease Multiomics Database [15]. A large cohort of IBD patients were included for this study. The stool samples of CD (*n* = 266), UC (*n* = 144), and non-IBD (*n* = 135) were collected. The extraction and purification steps have already been described previously [29]. The quality control (QC, *n* = 59) samples were also included. All clinical information from the samples is summarized in Table S2. The data format conversion and initial parameters were identical to the NIST dilution series above. The TICs of the data are shown in Figure S4. Parameter optimization was performed using four QC samples from each group randomly selected from the whole batch. The optimized parameters are provided in Table S1. The data analysis was finished with the whole MetaboAnalystR 3.0 workflow. Functional analysis was performed by integration with Mummichog2 for the comparisons between different groups (cutoff of *p* value 2.0 × 10^−6^).

## Figures and Tables

**Table 1:** Qualitative peak picking results of the different tools using different settings.

Methods	Total Peaks	True Peaks	Quantified Consensus	Gaussian Peak Ratio
Default	16,896	382	350	47.8%
IPO	24,346	744	663	52.0%
AutoTuner	25,517	664	603	40.5%
MetaboAnalystR 3.0	18,044	799	754	64.4%

True peaks are peaks that match the targeted metabolomics results with *m*/*z* ppm <10 and RT difference <0.3 min. Qualified consensus refers to the peaks where the relative error of intensity ratio between the two groups is less than 50% compared with the actual concentration. *Gaussian* Peak Ratio is the ratio of peaks with shapes following a *Gaussian* distribution (*cor* > 0.9 and *p* < 0.05).

**Table 2:** The pathway enrichment results (top 20, Crohn’s disease vs. non-IBD) generated by mummichog v1.0.8 and v2.0. Insignificant pathways (*p* value > 0.05) are shown in grey text.

Mummichog v1.0.8		Mummichog v2.0	
Pathways	*p* Value	Pathways	*p* Value
Bile acid biosynthesis	0.017199	Bile acid biosynthesis	0.011283
Vitamin D3 (cholecalciferol) metabolism	0.017526	Vitamin E metabolism	0.011321
Vitamin E metabolism	0.017966	Vitamin D3 (cholecalciferol) metabolism	0.014207
Carnitine shuttle	0.018084	Galactose metabolism	0.016026
Glycosphingolipid metabolism	0.021048	Glycerophospholipid metabolism	0.020464
De novo fatty acid biosynthesis	0.026554	Carnitine shuttle	0.021085
Keratan sulfate degradation	0.031317	Chondroitin sulfate degradation	0.025739
Fatty Acid Metabolism	0.032132	Vitamin B2 (riboflavin) metabolism	0.025739
N-Glycan Degradation	0.043912	Vitamin H (biotin) metabolism	0.025739
Phosphatidylinositol phosphate metabolism	0.053756	Fatty acid oxidation	0.025739
Hexose phosphorylation	0.069236	Omega-6 fatty acid metabolism	0.025739
Fatty acid activation	0.075044	Glycosphingolipid metabolism	0.041115
Limonene and pinene degradation	0.078492	Phosphatidylinositol phosphate metabolism	0.043604
Chondroitin sulfate degradation	0.082534	Hyaluronan Metabolism	0.04815
Glycosphingolipid biosynthesis - globoseries	0.082534	Putative anti-Inflammatory metabolites formation from EPA	0.04815
Saturated fatty acids beta-oxidation	0.082534	Electron transport chain	0.04815
Heparan sulfate degradation	0.082534	Heparan sulfate degradation	0.04815
Glycerophospholipid metabolism	0.09418	Sialic acid metabolism	0.061564
Starch and Sucrose Metabolism	0.13566	Vitamin A (retinol) metabolism	0.061564
Ascorbate (Vitamin C) and Aldarate Metabolism	0.14503	Saturated fatty acids beta-oxidation	0.061564

**Table 3:** Batch effect correction methods available in MetaboAnalystR 3.0.

Categories	Methods
QC Sample Independent	Combat [37], WaveICA [18], Eigens MS [38]
QC Sample Dependent	QC-RLSC [16], ANCOVA [39]
QC Metabolite Dependent	RUV-random [40], RUV2 [41], RUVseq [42]
Internal Standards Dependent	NOMIS [43], CCMN [44]

## Supplementary Materials

The following are available online at https://www.mdpi.com/2218-1989/10/5/186/s1, Figure S1: Bar plots of mummichog pathway enrichment results applied on Crohn’s disease patients versus non-IBD controls, Figure S2: Scatter plots of the mummichog pathway enrichment results applied on ulcerative colitis patients versus healthy controls, Figure S3: TICs of benchmark 1 (known standard data) before and after optimization, Figure S4: TICs of benchmark 2 (NIST series) before and after optimization, Figure S5: TICs of benchmark 3 (IBD data) before and after optimization, Table S1: Optimized parameters summary of all datasets, Table S2: Clinical characteristics summary of IBD subjects, Table S3: Mummichog (v.1) pathways (Top 20) of non-optimized IBD data (CD vs. non-IBD), Table S4: Mummichog (v.2) pathways of non-optimized IBD data (CD vs. non-IBD), Table S5: Mummichog (v.1) pathways (Top 20) of optimized IBD data (CD vs. non-IBD), Table S6: Mummichog (v.2) pathways (Top 20) of optimized IBD data (CD vs. non-IBD).
